# Associations between Polycyclic Aromatic Hydrocarbon–Related Exposures and *p53* Mutations in Breast Tumors

**DOI:** 10.1289/ehp.0901233

**Published:** 2009-11-18

**Authors:** Irina Mordukhovich, Pavel Rossner, Mary Beth Terry, Regina Santella, Yu-Jing Zhang, Hanina Hibshoosh, Lorenzo Memeo, Mahesh Mansukhani, Chang-Min Long, Gail Garbowski, Meenakshi Agrawal, Mia M. Gaudet, Susan E. Steck, Sharon K. Sagiv, Sybil M. Eng, Susan L. Teitelbaum, Alfred I. Neugut, Kathleen Conway-Dorsey, Marilie D. Gammon

**Affiliations:** 1 Department of Epidemiology, University of North Carolina, Chapel Hill, North Carolina, USA; 2 Department of Environmental Health Sciences, Mailman School of Public Health, Columbia University, New York, New York, USA; 3 Laboratory of Genetic Ecotoxicology, Institute of Experimental Medicine AS CR, Prague, Czech Republic; 4 Department of Epidemiology, Mailman School of Public Health, Columbia University, New York, New York, USA; 5 Department of Pathology, College of Physicians and Surgeons, Columbia University, New York, New York, USA; 6 Pathology Unit, Mediterranean Institute of Oncology, Catania, Italy; 7 Department of Epidemiology, Memorial Sloan-Kettering Cancer Center, New York, New York, USA; 8 Department of Epidemiology and Biostatistics, Arnold School of Public Health, University of South Carolina, Columbia, South Carolina, USA; 9 Department of Environmental Health Sciences, Harvard School of Public Health, Boston, Massachusetts, USA; 10 Epidemiologic Resources, Safety Evaluation, and Epidemiology, Pfizer, Inc., New York, New York, USA; 11 Department of Community and Preventive Medicine, Mount Sinai School of Medicine, New York, New York, USA; 12 Department of Medicine, College of Physicians and Surgeons, Columbia University, New York, New York, USA; 13 Lineberger Comprehensive Cancer Center, University of North Carolina–Chapel Hill, Chapel Hill, North Carolina, USA

**Keywords:** breast cancer, p53 mutation, p53 overexpression, PAH, polycylic aromatic hydrocarbons

## Abstract

**Background:**

Previous studies have suggested that polycyclic aromatic hydrocarbons (PAHs) may be associated with breast cancer. However, the carcinogenicity of PAHs on the human breast remains unclear. Certain carcinogens may be associated with specific mutation patterns in the *p53* tumor suppressor gene, thereby contributing information about disease etiology.

**Objectives:**

We hypothesized that associations of PAH-related exposures with breast cancer would differ according to tumor *p53* mutation status, effect, type, and number.

**Methods:**

We examined this possibility in a population-based case–control study using polytomous logistic regression. As previously reported, 151 *p53* mutations among 859 tumors were identified using Surveyor nuclease and confirmed by sequencing.

**Results:**

We found that participants with *p53* mutations were less likely to be exposed to PAHs (assessed by smoking status in 859 cases and 1,556 controls, grilled/smoked meat intake in 822 cases and 1,475 controls, and PAH–DNA adducts in peripheral mononuclear cells in 487 cases and 941 controls) than participants without *p53* mutations. For example, active and passive smoking was associated with *p53* mutation–negative [odds ratio (OR) = 1.55; 95% confidence interval (CI), 1.11–2.15] but not *p53* mutation–positive (OR = 0.77; 95% CI, 0.43–1.38) cancer (ratio of the ORs = 0.50, *p* < 0.05). However, frameshift mutations, mutation number, G:C→A:T transitions at CpG sites, and insertions/deletions were consistently elevated among exposed subjects.

**Conclusions:**

These findings suggest that PAHs may be associated with specific breast tumor *p53* mutation subgroups rather than with overall *p53* mutations and may also be related to breast cancer through mechanisms other than *p53* mutation.

Breast cancer is the second leading cancer-related cause of death among women in the United States (American Cancer Society 2008). Previous epidemiologic and experimental investigations suggest that polycyclic aromatic hydrocarbons (PAHs) may be associated with breast cancer (Bonner et al. 2005; el-Bayoumy et al. 1995; Gammon et al. 2002b, 2004b; Rundle et al. 2000). However, despite strongly positive associations in animal models and some evidence of a positive association in humans, the carcinogenicity of these chemical compounds on the human breast remains unclear.

PAHs are ubiquitous environmental pollutants formed by incomplete combustion of organic material (Samanta et al. 2002). These chemicals have estrogenic properties (Santodonato 1997), are known carcinogens in humans (Samanta et al. 2002), and cause mammary tumors in laboratory animals (el-Bayoumy et al. 1995; Hecht 2002). Exposure to PAHs in the general population occurs primarily through charred, smoked, and broiled foods; leafy vegetables (Phillips 1999); wood- and coal-burning stoves (Lewis et al. 1999); air pollution (Lioy and Greenberg 1990); and tobacco smoke (Besaratinia et al. 2002). PAH–DNA adducts (Gammon et al. 2004b), lifetime intake of grilled/smoked meat (Steck et al. 2007), and long-term passive smoking—but not current or former active smoking (Gammon et al. 2004a)—have been associated with breast cancer in our study population.

Cigarette smoke is associated with PAH–DNA adducts in human lymphocytes (Shantakumar et al. 2005), and the PAH benzo[*a*]pyrene (B[*a*]P) from cigarette smoke induces neoplastic transformation of human breast epithelial cells (Russo et al. 2002). However, smoking has been inconsistently linked to breast cancer in epidemiologic research, with more consistently positive findings reported for long-term passive smoking and among genetically susceptible subgroups (Ambrosone et al. 2008; Terry and Goodman 2006; Terry and Rohan 2002).

PAHs are formed on the surface of “well-done” meat (Kazerouni et al. 2001), but epidemiologic studies examining meat intake or doneness have yielded inconclusive results (Holmes et al. 2003; Zheng et al. 1998). These studies have primarily focused on recent dietary habits, whereas lifetime intake may be more relevant for carcinogenesis. Steck et al. (2007) observed a positive association between lifetime intake of grilled and smoked meat and breast cancer among postmenopausal women [middle vs. lowest tertile of intake, odds ratio (OR) = 1.47; 95% confidence interval (CI), 1.11–1.95; highest vs. lowest tertile of intake, OR = 1.47; 95% CI, 1.12–1.92)].

PAHs are metabolized through activation and detoxification pathways. When PAH exposure is high or detoxification is insufficient, PAH–DNA adducts form, including in breast tissue (Gammon and Santella 2008; Santella 1999). Adducts persist when repair mechanisms are inadequate (Braithwaite et al. 1999). Therefore, PAH–DNA adducts reflect both exposure to PAHs and host response—which differs because of variation in metabolism and/or DNA repair capacity between individuals—and are consistently associated with breast cancer in epidemiologic research (Gammon and Santella 2008).

The p53 protein is a transcription factor that regulates cell proliferation, differentiation, apoptosis, and DNA repair and therefore plays an important role in normal cell function and neoplastic transformation (Levine 1997). Certain carcinogens may be associated with specific mutation patterns in the *p53* tumor suppressor gene, and these characteristic patterns of DNA damage may contribute information about disease etiology by lending biologic support to exposure–disease associations and by helping to evaluate potential mechanisms of carcinogenesis (Greenblatt et al. 1994). Smoking has been associated with breast tumor *p53* mutational spectra (Conway et al. 2002). We hypothesized that associations between PAH-related exposures and breast cancer would differ according to tumor *p53* mutation status, effect, type, and number and we investigated this possibility using data from a population-based study.

## Materials and Methods

### Study population

Study subjects were participants from the Long Island Breast Cancer Study Project (LIBCSP), details of which have been published previously (Gammon et al. 2002a). Briefly, the LIBCSP is a population-based case–control study conducted among English-speaking women 20–98 years of age residing in Nassau and Suffolk counties in Long Island, New York. Cases were newly diagnosed with a first primary *in situ* or invasive breast cancer between 1996 and 1997, and were identified by rapid case ascertainment through contact with local pathology departments. Controls with no history of breast cancer were randomly selected from the same two counties using random digit dialing for women < 65 years of age and by Health Care Finance Administration rosters for women > 65 years of age. Controls were frequency matched to the expected age distribution of cases by 5-year age group. A total of 1,508 cases and 1,556 controls (82.1% and 62.7%, respectively, of all eligible subjects) completed the interview.

### Questionnaire assessment of PAH exposures

Trained interviewers completed in-home questionnaires with cases and controls. Questionnaire topics included reproductive history, annual household income, alcohol intake, race, education, active and passive cigarette smoking, lifetime dietary consumption of grilled and smoked foods, and other lifestyle factors. Ninety-eight percent of cases and 98% of controls completed a self-administered modified Block food frequency questionnaire (Block et al. 1986). These assessments were used to estimate lifetime intake of grilled and smoked meat, active and passive smoking, and daily intakes of B[*a*]P from meat and total energy using previously described methods (Gammon et al. 2004a; Steck et al. 2007).

### PAH–DNA adducts

Seventy-three percent of cases and 73% of controls donated a blood sample at the interview (Gammon et al. 2002a). DNA extracted from blood samples was used to assess PAH–DNA adduct levels in whole blood by competitive enzyme-linked immunosorbent assay (Gammon et al. 2002b). The antibody used in this analysis recognizes PAH diol epoxide adducts that form at the N^2^ position of guanine.

### p53 mutation analysis

Archived paraffin-embedded tumor tissue was obtained from participating hospitals for 67% of case participants. DNA was successfully extracted from tumor tissue for 859 women, and mutations were detected in exons 5–8 of *p53* through a multistep process (Rossner et al. 2008). Briefly, the samples were amplified using polymerase chain reaction (PCR), and the Surveyor Mutation Detection Kit (Transgenomic, Omaha, NE, USA) was used as a screening method for detection of *p53* mutations. Samples screening positive for potential mutations were selected for confirmation and identification of mutations by PCR amplification and sequencing using an ABI 3100 capillary sequencer (Applied Biosystems Inc., Foster City, CA, USA). Surveyor nuclease shows some single-base mismatch cutting preference, although this disadvantage may be ameliorated by formation of alternate heteroduplex mismatches (Qiu et al. 2004). As previously reported by Rossner et al. (2008), we identified a total of 151 *p53* mutations among 859 tumors, with 15 tumors harboring multiple mutations. In total, 15% of tumors contained one or more *p53* mutations, 83% of which were point mutations and 17% of which were insertions/deletions.

### p53 protein overexpression by immunohistochemistry

A total of 859 cases with available tumor tissue were evaluated for p53 protein expression by immunohistochemical staining using p53 mouse monoclonal antibody clone DO-1 (Immunotech, Inc., Westbrook, ME, USA) at 1:5 dilution (Rossner et al. 2008). We evaluated nuclear staining by a semiquantitative scoring system for intensity and percentage of positive nuclei. Tumors were considered positive if the staining had an intensity score of moderate or strong, if both study pathologists considered them positive, and if at least 10% of cells showed evidence of p53 protein expression. Previously published results from the LIBCSP show p53 overexpression in 36% of tumor samples (Rossner et al. 2008).

### Statistical methods

We used polytomous logistic regression (Hosmer and Lemeshow 1989) to estimate ORs and 95% CIs for associations between breast cancer categorized by *p53* tumor mutation subtype and smoking, intake of PAH food sources, and PAH–DNA adducts. We evaluated whether results for *p53* mutation–negative and *p53* mutation–positive tumors differed statistically by calculating ratios of the ORs for the two outcomes (Schlesselman 1982). Polytomous logistic regression (Hosmer and Lemeshow 1989) was also used to estimate the ORs and 95% CIs for associations between breast cancer categorized by tumor p53 protein expression status and these same PAH-related factors. Because cases and controls were frequency matched by 5-year age group, we adjusted all statistical models for age group at reference. We performed additional analyses to examine associations of active smoking, grilled and smoked meat intake, and a combined measure of these factors with *p53* mutation status, type, and effect using referent groups consisting of never smokers with low lifetime grilled and smoked meat intake.

We defined current smokers as women who smoked within 12 months of the reference date. Former smokers stopped smoking > 12 months before this date, and passive smokers were women who reported ever living with an active smoker (Gammon et al. 2004a). We categorized lifetime intake of grilled and smoked meat relative to the median intake of 4,160 lifetime servings among controls (Steck et al. 2007). Daily intake of B[*a*]P from meat was categorized relative to the median intake of 0.42 ng/day (Steck et al. 2007). PAH–DNA adducts were categorized as detectable or nondetectable; for analytical purposes, we categorized samples with < 15% inhibition as nondetectable (Gammon et al. 2002b).

We identified potential confounders by a thorough review of the relevant literature and analysis of causal diagrams (Shrier and Platt 2008). For PAH–DNA adducts, potential confounders were age at menarche, income, smoking status, and grilled and smoked meat intake. For dietary PAH intake, potential confounders were income, race, and energy intake. For smoking, potential confounders were income, alcohol intake, and education. We evaluated confounding by these covariates using backward selection with a 10% change in estimate criterion. In addition to adjustment for 5-year age group, we adjusted final models for daily alcohol intake when looking at smoking and age at menarche when looking at PAH–DNA adducts.

When examining associations with *p53* mutation status, participants were cases and controls with complete information regarding the exposure of interest and mutation status. As previously reported, the distribution of known and suspected breast cancer risk factors did not vary substantially between cases with and without available tumor tissue for *p53* mutation detection and p53 overexpression analysis (Rossner et al. 2008). Final sample sizes included 487 cases and 941 controls when examining PAH–DNA adducts, 859 cases and 1,556 controls for smoking status, and 822 cases and 1,475 controls for meat intake. Inclusion criteria and sample sizes were similar when examining associations with p53 protein expression status.

## Results

The distribution of *p53* mutations by smoking history, grilled and smoked meat intake, and PAH–DNA adducts is presented in Table 1, and associations between PAH-related exposures and *p53* mutation status are presented in Table 2. *p53* mutation–negative breast cancer was positively associated with detectable adducts (OR = 1.33; 95% CI, 1.02–1.73), active and passive smoking (OR = 1.55; 95% CI, 1.11–2.15), passive smoking from a spouse (OR = 1.25; 95% CI, 1.01–1.54), and high lifetime intake of grilled and smoked meat (OR = 1.51; 95% CI, 1.25–1.82). Effect estimates for all PAH-related exposures were elevated above the null for *p53* mutation–negative cancer.

In contrast, many PAH-related exposures were inversely associated with *p53* mutation–positive cancer (which primarily comprised missense mutations, but also included silent, nonsense, and frameshift mutations), and nearly all effect estimates for *p53* mutation–positive cancer for a given exposure were lower than the corresponding effect estimate for *p53* mutation–negative cancer (Table 2). Calculating ratios of the ORs (OR for the association between exposure and *p53* mutation–positive breast cancer divided by OR for the association between exposure and *p53* mutation–negative breast cancer) as an indicator of heterogeneity of ORs across groups yielded statistically significant differences when examining active and passive smoking exposure [*p53* mutation–positive cancer: OR = 1.55 (95% CI, 1.11–2.15); *p53* mutation– negative cancer: OR = 0.77 (95% CI, 0.43–1.38); ratio of the ORs, 0.50 (95% CI, 0.27–0.93)]. When we defined p53 status by protein expression status, we found no substantial heterogeneity in the ORs for the associations between PAH-related exposures and p53-positive cancer and the corresponding PAH exposures and p53-negative breast cancer [all *p*-values > 0.05 for the ratios of the ORs; see Supplemental Material, Table 3 (doi:10.1289/ehp.0901233)].

Associations between PAH-related exposures and selected *p53* mutation types are presented in Table 3. We were unable to statistically evaluate relations for all mutation types because of small numbers of certain mutations. We found consistently elevated associations between PAH-related exposures and G:C→A:T transitions at CpG sites as well as insertions/deletions. PAH-related exposures showed different directions of association with G:C→A:T transitions at non-CpG sites (Table 3), A:T→G:C transitions, and G:C→T:A transversions (data not shown). PAH exposures were also inconsistently related to *p53* missense, nonsense, and silent mutations (Table 4). Frameshift mutations were consistently associated with PAH-related exposures, and these associations were strongest for PAH–DNA adducts. Few effect estimates for mutation type or effect reached statistical significance.

Table 5 shows relations between PAH-related exposures and the number of tumor *p53* mutations. We found that many PAH-related exposures were associated with higher mutation number. For example, for detectable PAH–DNA adducts, ORs were 1.19 (95% CI, 0.63–2.25) for one mutation, 1.87 (95% CI, 0.38–9.20) for two mutations, and 2.15 (95% CI, 0.59–7.83) for three mutations relative to control participants, and ORs for high daily intake of B[*a*]Ps from meat were 1.17 (95% CI, 0.79–1.74) for one mutation, 1.79 (95% CI, 0.64–5.04) for two mutations, and 8.04 (95% CI, 2.29–28.27) for three mutations.

We also examined associations of *p53* mutation status with active smoking and lifetime intake of grilled and smoked meat using referent groups consisting of never smokers with low intake of grilled and smoked meat. Use of this approach strengthened associations between PAH-related factors and *p53* mutation–negative cancer, whereas associations with *p53* mutation–positive cancer were essentially unchanged [see Supplemental Material, Table 1 (doi:10.1289/ehp.0901233)]. In these analyses, *p53* mutation–negative cancer was associated with smoking history (ever vs. never; OR = 1.31; 95% CI, 1.03–1.67), current smoking (OR = 1.43; 95% CI, 1.06–1.93), and lifetime intake of grilled and smoked meat (OR = 1.61; 95% CI, 1.27–2.05).

Relations between PAH-related exposures and G:C→A:T transitions at CpG sites, insertions/deletions, and frameshift mutations were also strengthened by using the alternative referent groups. For example, examining associations between current smoking and insertions/deletions and between grilled and smoked meat intake and G:C→A:T transitions at CpG sites yielded ORs of 3.06 (95% CI, 0.87–10.77) and 2.68 (95% CI, 0.89–8.09), respectively [see Supplemental Material, Table 2 (doi:10.1289/ehp.0901233)]. We did not examine mutation number, A:T→G:C transitions, or G:C→T:A transversions due to low numbers of subjects with these mutations. Results were not altered substantially for missense, nonsense, and silent mutations (data not shown). Comparing current smokers who had high lifetime intake of grilled and smoked meat with never smokers who had low intake of grilled and smoked meat yielded ORs of 1.53 (95% CI, 1.07–2.20) for *p53* mutation–negative cancer, 4.24 (95% CI, 1.00–17.94) for G:C→A:T transitions at CpG sites, 4.49 (95% CI, 1.05–19.25) for insertions/deletions, and 4.43 (95% CI, 1.04–18.98) for frameshift mutations.

## Discussion

In this population-based analysis, we found that participants with breast tumor *p53* mutations were less likely to be exposed to PAH-related sources than were participants with *p53* mutation–negative cancer. However, frameshift mutations and number of mutations were consistently elevated in exposed subjects, and tumors of women exposed to PAH-related sources showed a pattern of increased G:C→A:T transitions at CpG sites and insertions/deletions. Associations of PAH-related exposures with *p53* mutation status, type, and effect were strengthened by minimizing PAH exposure in referent groups.

This is the first study to examine associations of dietary PAH intake, PAH–DNA adducts, and passive smoking with breast tumor *p53* mutations. It is the third study to examine associations between active smoking and breast cancer *p53* mutations, and has a larger sample size than the previous investigations (Conway et al. 2002; Van Emburgh et al. 2008). Few studies have looked at relations between exogenous exposures and *p53* mutations in breast cancer, although such research has potential to provide insights regarding breast cancer etiology.

We found a relatively low prevalence of *p53* mutations (15%) among our participants, which is consistent with the wide range reported in the literature (11–35%) (Goldman and Shields 1998; Tennis et al. 2006; Van Emburgh et al. 2008). This variation across studies may be due to differences between study populations, such as differences in distribution of age and race (Bowen et al. 2006; Klauber-DeMore 2005). Methodologic errors in mutation detection may also have contributed to the modest mutation prevalence. However, we selected a method previously shown to have a high sensitivity (Qiu et al. 2004) and that has been used successfully in various applications, including detection of *p53* mutations in hematologic malignancies (Mitani et al. 2007).

Immunohistochemical staining estimates p53 protein expression and is widely used as a proxy measure for detection of *p53* mutation status. However, the sensitivity of this method relative to mutation analysis is < 75% for breast cancer (Lacroix et al. 2006), and immunohistochemistry is subject to a number of methodologic limitations (Hall and McCluggage 2006; McCabe et al. 2005). We detected overexpression in 36% of tumor samples (Rossner et al. 2008), which is consistent with the range reported in the literature (30–40%) (Erdem et al. 2005; Iwase et al. 2001), but we did not find evidence of heterogeneity in the ORs for the associations between PAH-related exposures and p53-positive cancer as defined by protein expression status and the corresponding PAH exposures and p53-negative breast cancer. This is consistent with the results of one study of smoking and p53 overexpression (Furberg et al. 2002), and inconsistent with two other studies that noted an association between smoking and p53-positive breast cancer among younger women (Gammon et al. 1999; van der Kooy et al. 1996). Potential explanations for the discrepant findings across investigations include inability to identify specific *p53* mutational subtypes when using immunohistochemistry as a proxy, differences in metabolic activation and detoxification of PAHs between study populations, and chance findings.

Conway et al. (2002) found that current smokers were more likely and former smokers were less likely than never smokers to have *p53* mutation–positive breast cancer. In contrast, we found that both current and former smokers were less likely than never smokers to have *p53* mutation–positive cancer. Another epidemiologic study found positive, nonsignificant associations between smoking history and *p53* mutation prevalence (Van Emburgh et al. 2008). However, the number of *p53* mutation–positive cases (*n* = 34, mutation prevalence = 11%) was substantially smaller than in our study (*n* = 128, mutation prevalence = 15%) or in the study by Conway et al. (2002; *n* = 108, mutation prevalence = 24%), and the results appeared to be unstable. Reasons potentially underlying inconsistent results between studies include differences in age and race distributions and in metabolism and detoxification of PAHs between study populations, varying definitions of current smoking status, methodologic differences in mutation analysis, and chance findings.

The elevated associations between PAH-related exposures and *p53* mutation–negative cancer may indicate that PAH-related factors are related to breast cancer through mechanisms other than *p53* mutation. Grilled and smoked meat and tobacco smoke contain carcinogens other than PAHs, and PAH–DNA adducts or increased oxidative stress may lead to mutations in genes other than *p53*. Induction of DNA repair could also contribute to decreased mutation levels among women exposed to PAHs (Wei et al. 2000). *In vitro* and *in vivo* investigations indicate that repair is important in determining mutation patterns (Greenblatt et al. 1994). Furthermore, the fact that *p53* mutation patterns in tobacco-associated cancers show surprising variation is thought to be due at least partly to differences in DNA repair between cancer sites (Greenblatt et al. 1994). We found evidence of statistical heterogeneity in the ORs between *p53* mutation–positive and *p53* mutation–negative cancer when looking at exposure to both active and passive smoking. Associations between other PAH-related exposures and breast cancer did not differ statistically between mutation status subtypes, possibly due to insufficient power to detect such differences. Inverse associations between PAH exposures and *p53* mutation–positive cancer may be due to random variation around the null.

PAH-related exposures have been shown to increase both frameshift and base substitution mutations (Adonis and Gil 2000), and specific mutation effects may have etiologic associations. Relations of PAH exposures with nonsense and silent mutations were inconsistent in our study. Results for *p53* missense mutations were inconsistent as well, despite a larger sample size, with smoking and detectable adducts showing inverse associations and dietary PAH intake showing a positive association with missense mutations. It is plausible that different PAH-related exposures could lead to differing mutation effects, although this inconsistency may also be due to chance. Frameshift mutations were consistently elevated in exposed subjects, especially when looking at adducts and a combined measure of smoking and intake of grilled and smoked meat. PAHs induce frameshift mutations through adduct-induced deformation of the DNA helix (Greenblatt et al. 1994).

Participants with PAH-related exposures exhibited a fairly consistent mutational spectrum, characterized by increased proportions of insertions/deletions and G:C→A:T transitions at CpG sites. We found inconsistent results regarding G:C→A:T transversions and G:C→A:T transitions at non-CpG sites. This is in contrast to a previous study that reported that smokers showed an increased proportion of breast tumor G:C→T:A transversions and found some suggestion of decreased G:C→A:T transitions at CpG sites (Conway et al. 2002).

Previous research has suggested that the *p53* mutational spectrum of breast cancer includes high proportions of G:C→T:A transversions, G:C→A:T transitions at CpG sites, and insertions/deletions (Biggs et al. 1993; Goldman and Shields 1998; Greenblatt et al. 1994). The latter two mutation types are thought to result from endogenous processes (Greenblatt et al. 1994). However, it is possible that certain carcinogens produce a mutational spectrum similar to that due to endogenous processes (Biggs et al. 1993). For example, although benzo[*a*]pyrene diol epoxide adducts have been strongly associated with G→T transversions *in vitro* (Eisenstadt et al. 1982; Ruggeri et al. 1993), G→A transitions occur as the predominant mutation *in vivo* in some contexts because of influences of the local environment (Shukla et al. 1997). PAHs have been associated with transitions at G:C base pairs (Harris 1991), and transitions and frameshifts may be induced by PAH–DNA adducts (Greenblatt et al. 1994). PAHs may also cause DNA damage by generating reactive oxygen species, an endogenous mutagen (Singh et al. 2007). Moreover, it is very plausible that some carcinogens influence the rate of accumulation of mutations without altering the pattern (Biggs et al. 1993). The latter hypothesis is supported by our finding that likelihood of PAH exposure increases with tumor *p53* mutation number.

A number of studies found results similar to ours when examining associations between PAH-related sources and *p53* mutations in smoking-related cancers (Diergaarde et al. 2003; Fryzek et al. 2006; Harty et al. 1996; Schroeder et al. 2003; Zhang et al. 2006). Most studies that examined *p53* mutational spectra found a difference in spectrum by exposure status, despite null or inverse associations with *p53* mutation prevalence, possibly because associations with specific mutation patterns were washed out when all mutations were combined into a single case subgroup. For example, a study by Schroeder et al. (2003) that observed no difference in *p53* mutation frequency in bladder cancer according to smoking status found that smoking was strongly associated with CpG G:C→A:T transitions.

The main limitation of our study is the sample size for examining subgroup mutation effects, for example, by type or number. Some of our point estimates have very wide CI estimates, and these results should be interpreted with caution. We cannot rule out that our findings are due to chance. However, this is the largest study regarding PAH-related exposures and breast tumor *p53* mutations conducted to date, and the first study to look at associations for most of the PAH-related exposures examined in this investigation. Thus, we believe that our analysis is an important addition to the sparse literature for this topic. The strengthened associations observed after minimizing PAH exposure in referent groups provide further support for a relation between PAHs and breast cancer *p53* mutation status, type, and effect.

A potential limitation in any retrospective, case–control analysis is the possibility of recall bias. However, cases were unaware of the mutation patterns in their tumor tissue, so this potential bias is less likely to affect case–case comparisons. Many of the exposure variables examined were surrogates of PAH exposure, although PAH–DNA adducts are an internal measure of PAH exposure and response (Gammon and Santella 2008). However, PAH–DNA adducts reflect only recent exposures, whereas exposures in the more distant past are likely to be relevant to carcinogenesis. Evaluation of exons 5–8 only is likely to underestimate the prevalence of *p53* mutations. However, these exons contain > 90% of mutations reported in breast cancer (Lacroix et al. 2006). Missense mutations are concentrated in the central part of *p53*, whereas nonsense, silent, and frameshift mutations are distributed throughout the coding region (Greenblatt et al. 1994).

Previous research has suggested that associations between PAHs and *p53* mutation pattern may vary by genetic variants for detoxification and DNA repair enzymes (Biggs et al. 1993; Schroeder et al. 2003; Van Emburgh et al. 2008) and that the carcinogenic effects of PAHs on the breast may be stronger among women with low antioxidant intake (Steck et al. 2007). Therefore, future high-powered analyses should examine effect modification by genotypes relevant to detoxification, antioxidative response, and DNA repair and by antioxidant intake. Future studies should also categorize exposures more finely and should assess the importance of timing of PAH exposure on *p53* mutagenesis.

## Conclusions

Our findings suggest that PAH exposures may be associated with breast tumor *p53* mutation effect, type, and number rather than with overall *p53* mutations, and may also be related to breast cancer through mechanisms other than *p53* mutation. The high incidence of breast cancer implies that even modestly increased risks for tumor subgroups may have substantial public health significance. This analysis has extended previous research regarding a sparsely investigated topic. Our results provide new information to guide future research regarding the carcinogenic effects of PAH exposure on the human breast.

## Figures and Tables

**Table 1:** Distribution of *p53* mutations by PAH-related exposures among LIBCSP case participants [*n* (%)].

	Cigarette smoking history (*n* = 859)		Grilled and smoked meat intake^a^ (*n* = 822)		Detectable PAH–DNA adducts (*n* = 487)	
Mutation	Ever	Never	High	Low	Yes	No
Point mutations						
Transitions						
G:C→A:T at CpG	23 (29.1)	12 (16.7)	22 (28.2)	12 (17.1)	16 (27.1)	5 (23.8)
G:C→A:T at non-CpG	19 (24.1)	27 (37.5)	19 (24.4)	27 (38.6)	17 (28.8)	7 (33.3)
A:T→G:C	7 (8.9)	8 (11.1)	6 (7.7)	9 (12.9)	7 (11.9)	3 (14.3)
Transversions						
G:C→T:A	5 (6.3)	12 (16.7)	9 (11.5)	6 (8.6)	5 (8.5)	1 (4.8)
G:C→C:G	5 (6.3)	1 (1.4)	4 (5.1)	2 (2.9)	1 (1.7)	1 (4.8)
A:T→T:A	3 (3.8)	2 (2.8)	2 (2.6)	3 (4.3)	1 (1.7)	2 (9.5)
A:T→C:G	0 (0)	1 (1.4)	0 (0)	1 (1.4)	0 (0)	0 (0)
Insertions or deletions	17 (21.5)	9 (12.5)	16 (20.5)	10 (14.3)	12 (20.3)	2 (9.5)
Base change						
C→T at CpG	13 (21.0)	6 (9.5)	12 (19.4)	6 (10.0)	11 (23.4)	2 (10.5)
G→A at CpG	10 (16.1)	6 (9.5)	10 (16.1)	6 (10.0)	5 (10.6)	3 (15.8)
C→T at non-CpG	11 (17.7)	20 (31.8)	15 (24.2)	16 (26.7)	12 (25.5)	4 (21.1)
G→A at non-CpG	8 (12.9)	7 (11.1)	4 (6.5)	11 (18.3)	5 (10.6)	3 (15.8)
T→C	3 (4.8)	5 (7.9)	3 (4.8)	5 (8.3)	3 (6.4)	1 (5.3)
A→G	4 (6.5)	3 (4.8)	3 (4.8)	4 (6.7)	4 (8.5)	2 (10.5)
G→T	1 (1.6)	11 (17.5)	6 (9.7)	5 (8.3)	2 (4.3)	1 (5.3)
C→A	4 (6.5)	1 (1.6)	3 (4.8)	1 (1.7)	3 (6.4)	0 (0)
G→C	3 (4.8)	1 (1.6)	3 (4.8)	1 (1.7)	0 (0)	1 (5.3)
C→G	2 (3.2)	0 (0)	1 (1.6)	1 (1.7)	1 (2.1)	0 (0)
T→A	1 (1.6)	2 (3.2)	0 (0)	3 (5.0)	1 (2.1)	1 (5.3)
A→T	2 (3.2)	0 (0)	2 (3.2)	0 (0)	0 (0)	1 (5.3)
T→G	0 (0)	1 (1.6)	0 (0)	1 (1.7)	0 (0)	0 (0)
Mutation effect						
Missense	38 (48.1)	42 (58.3)	41 (52.6)	36 (51.4)	23 (39.0)	18 (85.7)
Nonsense	8 (10.1)	8 (11.1)	7 (9.0)	9 (12.9)	6 (10.2)	1 (4.8)
Silent	16 (20.3)	13 (18.1)	14 (18.0)	15 (21.4)	18 (30.5)	0 (0)
Frameshift	17 (21.5)	9 (12.5)	16 (20.5)	10 (14.3)	12 (20.3)	2 (9.5)
No. of mutations	79	72	78	70	59	21
Tumors with mutations	66 (13.8)	62 (16.4)	63 (13.2)	62 (17.9)	47 (13.0)	18 (14.4)
Tumors with no mutations	414 (86.3)	317 (83.6)	413 (86.8)	284 (82.1)	315 (87.0)	107 (85.6)
Total no. of tumors	480	379	476	346	362	125

a Lifetime intake of grilled and smoked meat is dichotomized based on median lifetime servings among controls (median = 4,160 servings).

**Table 2:** Associations between PAH-related exposures and the risk of breast cancer subtype as defined by *p53* mutation status in the LIBCSP.^a^

		OR (95% CI)	
Mutation status	Cases/controls (*n*)	Age-adjusted	Multivariate-adjusted
PAH–DNA adducts (detectable vs. nondetectable)			
*p53*+ breast cancer	65/941	1.16 (0.66–2.04)	1.28 (0.71–2.31)
*p53*− breast cancer	422/941	1.34 (1.03–1.75)	1.33 (1.02–1.73)
Ratio of the ORs (*p53*+ vs. *p53*−)		0.86 (0.48–1.55)	0.96 (0.52–1.78)

Ever active smoking versus never active smoking			
*p53*+ breast cancer	128/1,556	0.90 (0.62–1.30)	0.96 (0.66–1.39)
*p53*− breast cancer	731/1,556	1.11 (0.92–1.32)	1.09 (0.91–1.31)
Ratio of the ORs (*p53*+ vs. *p53*−)		0.82 (0.56–1.19)	0.88 (0.60–1.29)

Current active smoking versus never active smoking			
*p53*+ breast cancer	80/989	0.80 (0.46–1.40)	0.87 (0.50–1.54)
*p53*− breast cancer	468/989	1.22 (0.96–1.56)	1.21 (0.94–1.55)
Ratio of the ORs (*p53*+ vs. *p53*−)		0.66 (0.37–1.17)	0.72 (0.40–1.29)

Past active smoking versus never active smoking			
*p53*+ breast cancer	110/1,262	0.97 (0.65–1.44)	1.03 (0.69–1.55)
*p53*− breast cancer	580/1,262	1.06 (0.86–1.29)	1.05 (0.85–1.29)
Ratio of the ORs (*p53*+ vs. *p53*−)		0.91 (0.60–1.39)	0.99 (0.65–1.51)

Both active and passive smoking versus never passive or active smoking			
*p53*+ breast cancer	68/875	0.71 (0.40–1.25)	0.77 (0.43–1.38)
*p53*− breast cancer	417/875	1.58 (1.14–2.18)	1.55 (1.11–2.15)
Ratio of the ORs (*p53*+ vs. *p53*−)		0.45 (0.24–0.82)	0.50 (0.27–0.93)

Active smoking only versus never passive or active smoking			
*p53*+ breast cancer	35/328	1.14 (0.55–2.38)	1.23 (0.58–2.62)
*p53*− breast cancer	129/328	1.33 (0.86–2.04)	1.44 (0.93–2.24)
Ratio of the ORs (*p53*+ vs. *p53*−)		0.86 (0.39–1.87)	0.86 (0.39–1.90)

Ever passive smoking only versus never passive or active smoking			
*p53*+ breast cancer	61/681	0.90 (0.50–1.61)	0.94 (0.52–1.68)
*p53*− breast cancer	306/681	1.38 (0.99–1.91)	1.38 (0.99–1.91)
Ratio of the ORs (*p53*+ vs. *p53*−)		0.65 (0.35–1.21)	0.68 (0.37–1.27)

Ever passively exposed to spouse versus never passively exposed to spouse			
*p53*+ breast cancer	93/1,228	1.64 (1.03–2.60)	1.64 (1.03–2.61)
*p53*− breast cancer	602/1,228	1.25 (1.01–1.54)	1.25 (1.01–1.54)
Ratio of the ORs (*p53*+ vs. *p53*−)		1.31 (0.81–2.12)	1.32 (0.82–2.13)

Lifetime intake of smoked/grilled meat (high vs. low)^b^			
*p53*+ breast cancer	125/1,475	1.08 (0.74–1.57)	1.08 (0.74–1.57)
*p53*− breast cancer	697/1,475	1.51 (1.25–1.82)	1.51 (1.25–1.82)
Ratio of the ORs (*p53*+ vs. *p53*−)		0.72 (0.48–1.06)	0.72 (0.48–1.06)

Total B[*a*]Ps from meat (high intake vs. low intake)^c^			
*p53*+ breast cancer	124/1,473	1.28 (0.88–1.87)	1.28 (0.88–1.87)
*p53*− breast cancer	700/1,473	1.04 (0.86–1.25)	1.04 (0.86–1.25)
Ratio of the ORs (*p53*+ vs. *p53*−)		1.24 (0.84–1.83)	1.24 (0.84–1.83)

a In addition to adjustment for age group, final models were adjusted for daily alcohol intake when examining smoking exposure and age at menarche when examining PAH–DNA adducts. Ratios of the ORs [OR for the association between exposure and *p53*-positive (*p53*+) breast cancer divided by OR for the association between exposure and *p53*-negative (*p53*−) breast cancer] were calculated as indicators of heterogeneity of effects across groups.

b Lifetime intake of grilled and smoked meat is dichotomized based on median lifetime servings among controls (median, 4,160 servings).

c Daily intake of B[*a*]Ps from meat is dichotomized based on median daily intake among controls (median, 0.42 ng/day).

**Table 3:** Associations between PAH-related exposures and the risk of breast cancer subtype as defined by *p53* mutation type in the LIBCSP.^a^

		OR (95% CI)	
Mutation type	Cases/controls (*n*)	Age adjusted	Multivariate adjusted
G:C→A:T at CpG transitions			
PAH–DNA adducts			
Nondetectable	5/293	1.0	1.0
Detectable	16/648	1.51 (0.55–4.19)	1.53 (0.55–4.23)
Smoking status			
Never	12/698	1.0	1.0
Former	16/564	1.60 (0.74–3.44)	1.65 (0.76–3.58)
Current	6/291	1.33 (0.49–3.67)	1.53 (0.55–4.27)
Grilled and smoked meat^b^			
Low lifetime intake	12/739	1.0	1.0
High lifetime intake	21/736	1.69 (0.82–3.52)	1.69 (0.82–3.52)

G:C→A:T at non-CpG transitions			
PAH–DNA adducts			
Nondetectable	6/293	1.0	1.0
Detectable	15/648	1.10 (0.42–2.89)	1.09 (0.41–2.85)
Smoking status			
Never	24/698	1.0	1.0
Former	14/564	0.73 (0.37–1.44)	0.80 (0.40–1.58)
Current	4/291	0.44 (0.15–1.29)	0.51 (0.17–1.52)
Grilled and smoked meat			
Low intake	25/739	1.0	1.0
High intake	17/736	0.72 (0.38–1.37)	0.72 (0.38–1.37)

Insertions/deletions			
PAH–DNA adducts			
Nondetectable	2/293	1.0	1.0
Detectable	12/648	2.64 (0.58–11.91)	4.77 (0.63–36.25)
Smoking status			
Never	9/698	1.0	1.0
Former	10/564	1.45 (0.58–3.65)	1.44 (0.56–3.69)
Current	7/291	2.11 (0.75–5.95)	2.41 (0.85–6.85)
Grilled and smoked meat			
Low intake	10/739	1.0	1.0
High intake	16/736	1.68 (0.74–3.78)	1.68 (0.74–3.78)

a In addition to adjustment for age group, final models were adjusted for daily alcohol intake when examining smoking exposure and age at menarche when examining PAH–DNA adducts.

b Lifetime intake of grilled and smoked meat is dichotomized based on median lifetime servings among controls (median, 4,160 servings).

**Table 4:** Associations between PAH-related exposures and the risk of breast cancer subtype as defined by *p53* mutation effect in the LIBCSP.^a^

		OR (95% CI)	
Mutation effect	Cases/controls (*n*)	Age adjusted	Multivariate adjusted
Missense mutations			
PAH–DNA adducts			
Nondetectable	15/293	1.0	1.0
Detectable	20/648	0.62 (0.31–1.24)	0.67 (0.33–1.36)
Active smoking status			
Never	37/698	1.0	1.0
Former	26/564	0.87 (0.52–1.47)	0.92 (0.54–1.56)
Current	7/291	0.51 (0.22–1.18)	0.53 (0.23–1.24)
Grilled/smoked meat^b^			
Low lifetime intake	33/739	1.0	1.0
High lifetime intake	34/736	1.09 (0.66–1.80)	1.09 (0.66–1.80)

Nonsense mutations			
PAH–DNA adducts			
Nondetectable	1/293	1.0	1.0
Detectable	6/648	2.57 (0.31–21.47)	2.60 (0.31–21.94)
Active smoking status			
Never	8/698	1.0	1.0
Former	7/564	1.05 (0.37–2.94)	1.21 (0.42–3.48)
Current	1/291	0.39 (0.05–3.28)	0.42 (0.05–3.56)
Grilled/smoked meat			
Low intake	9/739	1.0	1.0
High intake	7/736	0.84 (0.30–2.33)	0.84 (0.30–2.33)

Silent mutations			
PAH–DNA adducts			
Nondetectable	0/293	1.0	1.0
Detectable	15/648	—	—
Active smoking status			
Never	11/698	1.0	1.0
Former	9/564	1.01 (0.41–2.48)	1.17 (0.47–2.90)
Current	4/291	1.15 (0.35–3.78)	1.37 (0.42–4.54)
Grilled/smoked meat			
Low intake	15/739	1.0	1.0
High intake	9/736	0.65 (0.28–1.53)	0.65 (0.28–1.53)

Frameshift mutations			
PAH–DNA adducts			
Nondetectable	2/293	1.0	1.0
Detectable	12/648	2.62 (0.58–11.80)	4.85 (0.62–37.75)
Active smoking status			
Never	9/698	1.0	1.0
Former	10/564	1.44 (0.57–3.63)	1.43 (0.56–3.66)
Current	7/291	2.11 (0.75–5.96)	2.40 (0.84–6.85)
Grilled/smoked meat			
Low intake	10/739	1.0	1.0
High intake	16/736	1.68 (0.75–3.79)	1.68 (0.75–3.79)

a In addition to adjustment for age group, final models were adjusted for daily alcohol intake when examining smoking exposure and age at menarche when examining PAH–DNA adducts.

b Lifetime intake of grilled and smoked meat is dichotomized based on median lifetime servings among controls (median, 4,160 servings).

**Table 5:** Associations between PAH-related exposures and the number of tumor *p53* mutations relative to control participants in the LIBCSP.^a^

		OR (95% CI)	
No. of mutations	Cases/controls (*n*)	Age adjusted	Multivariate adjusted
PAH–DNA adducts (detectable vs. nondetectable)			
1	55/941	1.06 (0.58–1.94)	1.19 (0.63–2.25)
2	10/941	1.85 (0.38–9.01)	1.87 (0.38–9.20)
3	15/941	2.10 (0.58–7.58)	2.15 (0.59–7.83)

Ever active smoking vs. never active smoking			
1	113/1,556	0.89 (0.61–1.32)	0.94 (0.63–1.40)
2	16/1,556	0.84 (0.31–2.28)	0.98 (0.35–2.72)
3	18/1,556	0.96 (0.38–2.46)	1.13 (0.44–2.92)

Current active smoking vs. never active smoking			
1	72/989	0.83 (0.47–1.47)	0.90 (0.51–1.62)
2	10/989	0.83 (0.16–4.23)	0.88 (0.17–4.57)
3	9/989	—	—

Past active smoking vs. never active smoking			
1	96/1,262	0.93 (0.61–1.43)	0.99 (0.64–1.52)
2	14/1,262	0.96 (0.32–2.86)	1.08 (0.36–3.30)
3	18/1,262	1.36 (0.53–3.50)	1.60 (0.61–4.18)

Both active and passive smoking vs. never passive or active smoking			
1	60/875	0.65 (0.36–1.17)	0.69 (0.37–1.27)
2	8/875	0.72 (0.14–3.71)	0.87 (0.17–4.61)
3	9/875	—	—

Active smoking only vs. never passive or active smoking			
1	33/328	1.14 (0.53–2.42)	1.23 (0.57–2.66)
2	4/328	1.39 (0.19–10.31)	1.50 (0.20–11.38)
3	0/328	—	—

Ever passive smoking only vs. never passive or active smoking			
1	54/681	0.78 (0.42–1.42)	0.81 (0.44–1.49)
2	8/681	1.71 (0.32–9.21)	1.73 (0.32–9.38)
3	9/681	—	—

Ever passively exposed to spouse vs. never passively exposed to spouse			
1	80/1,228	1.50 (0.92–2.46)	1.51 (0.92–2.46)
2	12/1,228	2.15 (0.61–7.56)	2.21 (0.63–7.70)
3	18/1,228	3.36 (0.94–12.04)	3.23 (0.90–11.57)

Lifetime intake of smoked/grilled meat (high vs. low)^b^			
1	110/1,475	0.98 (0.66–1.46)	0.98 (0.66–1.46)
2	16/1,475	3.64 (1.12–11.79)	3.64 (1.12–11.79)
3	18/1,475	1.16 (0.45–2.99)	1.16 (0.45–2.99)

Total B[*a*]Ps from meat (high intake vs. low intake)^c^			
1	109/1,475	1.17 (0.79–1.74)	1.17 (0.79–1.74)
2	16/1,475	1.79 (0.64–5.04)	1.79 (0.64–5.04)
3	18/1,475	8.04 (2.29–28.27)	8.04 (2.29–28.27)

—, insufficient sample size.

a In addition to adjustment for age group, final models were adjusted for daily alcohol intake when examining smoking exposure and age at menarche when examining PAH–DNA adducts.

b Lifetime intake of grilled and smoked meat is dichotomized based on median lifetime servings among controls (median, 4,160 servings).

c Daily intake of B[*a*]Ps from meat is dichotomized based on median daily intake among controls (median, 0.42 ng/day).
